# Acute Malnutrition Among Children, Mortality, and Humanitarian Interventions in Conflict-Affected Regions — Nigeria, October 2016–March 2017

**DOI:** 10.15585/mmwr.mm6648a4

**Published:** 2017-12-08

**Authors:** Eva Leidman, Erin Tromble, Adamu Yermina, Robert Johnston, Chris Isokpunwu, Adeyemi Adeniran, Assaye Bulti

**Affiliations:** ^1^Division of Global Health Protection, Center for Global Health, CDC; ^2^Epidemic Intelligence Service, CDC; ^3^Nutrition Section, United Nations Children Fund, Abuja, Nigeria; ^4^Nutrition Division, Federal Ministry of Health, Abuja, Nigeria; ^5^Real Sector and Household Statistics, National Bureau of Statistics, Abuja, Nigeria.

A public health emergency was declared by the Nigerian Federal Ministry of Health in northeastern Nigeria in June 2016 and escalated by the United Nations to a Level 3 Emergency in August 2016, after confirmation of wild poliovirus and measles outbreaks and evidence that prevalence of acute malnutrition exceeded emergency thresholds in areas newly liberated from Boko Haram control (*1*,*2*). To monitor rates of mortality, acute malnutrition among children, infectious disease morbidity, and humanitarian interventions after the emergency declaration, a series of cross-sectional household surveys were conducted in fall 2016 and winter 2017 in the northeastern states of Borno and Yobe using a cluster methodology. All-cause mortality among all age groups (crude mortality) and among children aged <5 years (under-five mortality) were above emergency thresholds in 2017 and significantly increased from 2016, despite evidence of increased preventive public health interventions, including measles vaccination. Access to treatment for common childhood illnesses remained very low, as evidenced by reports of fewer than one in six children in areas outside Borno’s capital receiving any care for diarrhea. The data from these surveys provide evidence of excessively high mortality (particularly among children), highlight the impact of ongoing violence, and underscore the need for humanitarian efforts to scale up access to treatment services in conflict-affected areas.

After the emergency declarations, the Nigerian National Bureau of Statistics, in coordination with the National Population Commission, the Federal Ministry of Health, United Nations Children’s Fund, and CDC collaborated to conduct a series of two-stage cluster surveys to assess mortality, malnutrition, and access to and receipt of essential public health services. This report presents findings from the first two rounds of data collection (October 11–November 17, 2016, and February 13–March 29, 2017).

Enumeration areas from the national sampling frame were used as clusters for Yobe. Because of ongoing displacement in Borno, clusters there included both settled villages and camps of internally displaced persons. Within each state, local government areas were grouped into substate regions based on geography and conflict impact. Data were stratified by region (Borno: Southern, Central, and the Borno capital area comprising Maiduguri and Jere; Yobe: Central, Southern, and Northern). Clusters for each region were drawn independently, with probability of selection proportional to size. Within selected clusters, households were selected applying systematic random selection. Areas that were known to be inaccessible because of security concerns were excluded. Adjusting for nonresponse, a sample size of 1,800 households was calculated for each of the two states in the first round. In the second round, 1,860 households were included for Borno, and the Southern Yobe region was oversampled to allow for disaggregated analysis of areas with greater conflict, yielding a target sample of 2,480 households in Yobe. All age-eligible children were measured using standard anthropometric procedures (*3*). Nutritional status was classified using 2006 World Health Organization (WHO) growth standards (*4*). Pearson's chi-square test was used to determine significant differences (p<0.05) between rounds. This project was reviewed in accordance with CDC human subjects protection procedures and was determined to be nonresearch and not subject to review by an institutional review board.

The final sample in Borno comprised 1,719 households (including 1,557 children aged 0–59 months) in the first round and 1,729 households (1,813 children) in the second. In the first and second rounds in Yobe, 1,667 households (1,692 children) and 2,393 households (2,729 children), respectively, were selected. Among selected clusters, 13 (7.2%) in the first round and 15 (6.9%) in the second round were inaccessible or abandoned. Household response rates in the accessed clusters exceeded 96% in all regions in both rounds.

Prevalence of global acute malnutrition (weight-for-height z-scores less than -2 or bilateral pitting edema) among children aged 0–59 months was significantly higher in the first round than the second round in Maiduguri and Jere (13.0% compared with 6.4%) and in both Southern (10.7% compared with 7.8%) and Northern Yobe (14.3% compared with 8.6%). During the first round, prevalence of global acute malnutrition ranged from 8.9% to 14.3% by region and from 6.4% to 8.6% in the second round (Table 1). Crude and under-five mortality rates increased from the first to the second round in all regions. The increase in crude mortality was significant in two Borno regions (Southern and Central) and two Yobe regions (Southern and Northern) (p<0.05). The under-five mortality rate was approximately three times higher than the crude mortality rate in Southern Borno for both rounds and during the first round in Central Borno and Central and Northern Yobe (Table 1). Crude and under-five mortality both exceeded the emergency thresholds of one and two deaths per 10,000 per day, respectively, in Central Borno as well as Central and Northern Yobe during the second round. The under-five morality rate in Southern Yobe also exceeded the emergency threshold.

**TABLE 1:** Emergency survey prevalence of acute malnutrition among children and crude and under-five mortality rates, by region — Northeastern Nigeria, round 1 (October–November 2016) and round 2 (February–March 2017)

State/Region	Acute malnutrition, by weight-for-height, children aged 0–59 mos				Crude mortality^§^		Under-five mortality^¶^	
	Global acute malnutrition*		Severe acute malnutrition^†^					
	Round 1	Round 2	Round 1	Round 2	Round 1	Round 2	Round 1	Round 2
	% (95% CI)	% (95% CI)	% (95% CI)	% (95% CI)	Rate** (95% CI)	Rate** (95% CI)	Rate** (95% CI)	Rate** (95% CI)
**Borno**								
Southern Borno	8.9 (6.7–11.0)	6.4 (4.0–10.2)	1.1 (0.5–2.8)	0.8 (0.3–2.2)	0.26 (0.17–0.41)	0.56 (0.25–1.22)	0.97 (0.56–1.6)	1.96 (0.84–4.49)
Central Borno	11.6 (8.8–15.2)	7.8 (4.8–12.4)	0.6 (0.2–1.8)	1.2 (0.5–2.8)	0.55 (0.35–0.85)^††^	1.36^§§^ (0.83–2.20)^††^	1.69 (0.96–2.91)	2.60^§§^ (1.62–4.12)
Maiduguri and Jere	13.0 (10.2–16.4)^††^	6.4 (4.6–8.9)^††^	1.3 (0.6–2.9)	0.8 (0.3– 2.1)	0.30 (0.16–0.57)^††^	0.85 (0.53–1.36)^††^	0.78 (0.34–1.78)	1.45 (0.76–2.74)
**Yobe**								
Central Yobe	10.3 (7.3–14.2)	8.1 (5.8–11.3)	2.1 (1.1–4.2)	0.9 (0.5–1.8)	0.63 (0.39–1.01)	1.14^§§^ (0.81–1.61)	2.06^§§^ (1.24–3.38)	2.49^§§^ (1.61–3.82)
Southern Yobe	10.7 (8.3–13.6)^††^	7.8 (6.3–9.6)^††^	1.6 (0.8–3.0)	0.8 (0.4–1.6)	0.36 (0.24–0.54)^††^	0.91 (0.59–1.24)^††^	0.90 (0.56–1.67)	2.17^§§^ (1.13–3.21)
Northern Yobe	14.3 (10.6–18.9)^††^	8.6 (6.2–11.9)^††^	1.6 (0.7–3.9)	1.0 (0.5–2.0)	0.50 (0.36–0.68)^††^	1.02^§§^ (0.70–1.47)^††^	1.69 (0.96–2.91)	2.63^§§^ (1.54–4.43)

**Abbreviation:** CI = confidence interval.

* Weight-for-height z-scores less than -2 or bilateral pitting edema.

^†^ Weight-for-height z-scores less than-3 or bilateral pitting edema.

^§^ Mortality rate among all age groups from all causes.

^¶^ Mortality rate among children aged 0–59 months.

** Rates reported as deaths per 10,000 population per day.

^††^ Statistically significant difference between round 1 and round 2.

^§§^ Rate exceeded emergency thresholds (1 per 10,000 per day for crude mortality and 2 per 10,000 per day for mortality among children aged <5 years).

Use of public health interventions was assessed using the following four indicators: 1) measles vaccination coverage, 2) receipt of one or more services through a public health outreach campaign, 3) receipt of anthelmintic prophylaxis in the 6 months preceding the survey, and 4) receipt of fortified cereals (Table 2). The proportion of children aged 12–59 months that had received at least 1 dose of a measles-containing vaccine, as determined by either vaccination card or parental recall, was <65% in all regions in the first round (range = 28.8%–63.5%). Measles vaccination coverage increased significantly in the second round in all regions except Southern Borno, with the greatest absolute increases in Central Borno (from 33.7% to 78.9%) and Northern Yobe (from 28.8% to 67.1%). No region attained the 95% vaccination coverage threshold necessary to establish herd immunity. The proportion of households reporting receipt of one or more of the maternal, neonatal, and child health services (such as vaccinations, nutritional screening, birth registration, and bed-net distributions) during a government sponsored public health outreach campaign was <10% in all regions except Southern Borno (11.9%) during the first round of surveys. The percentage of children aged 12–59 months who received anthelmintic prophylaxis during the 6 months preceding the survey was also <10% in all regions (range = 2.9%–6.2%) during the first round. Increases in the receipt of services through the outreach campaign (range = 11.7%–26.6%) and of anthelmintic prophylaxis use (range = 7.8%–21.1%) were significant in all regions during the second round of surveys. However, no increase was observed in the distributions of fortified cereals.

**TABLE 2:** Emergency survey coverage with measles vaccination, public health outreach campaigns, anthelmintic medication, and distribution of fortified cereals, by region — Northeastern Nigeria, round 1 (October–November 2016) and round 2 (February–March 2017)

State/Region	Measles vaccination coverage, by recall or vaccination card, among children aged 12–59 mos		Coverage with the preceding public health outreach campaign, among all households		6-month coverage of anthelmintic medication, among children aged 12–59 mos		Receipt of fortified cereals in the last 6 months, among all households	
	Round 1	Round 2	Round 1	Round 2	Round 1	Round 2	Round 1	Round 2
	% (95% CI)	% (95% CI)	% (95% CI)	% (95% CI)	% (95% CI)	% (95% CI)	% (95% CI)	% (95% CI)
**Borno**								
Southern Borno	57.1 (41.7–71.3)*	76.9 (64.2–86.1)*	11.9 (5.7–23.1)	38.5 (28.2–49.9)	2.9 (1.2–6.9)	19.3 (11.9–29.8)	0.2 (0.0–1.2)*	0.5 (0.2–1.5)*
Central Borno	33.7 (22.7–46.7)	78.9 (66.1–87.7)	6.5 (3.0–13.6)	27.4 (17.2–40.7)	3.5 (1.2–9.6)	15.2 (7.9–27.1)	1.7 (0.4–6.8)*	1.7 (0.7–3.8)*
Maiduguri and Jere	63.5 (52.4–73.4)	83.9 (77.3–88.9)	8.7 (4.1–17.8)	29.5 (19.8–41.5)	4.7 (2.3–9.4)	17.2 (10.5–26.9)	1.5 (0.4–5.1)*	3.5 (1.2–10.0)*
**Yobe**								
Central Yobe	34.1 (24.4–45.3)	67.1 (52.4–79.1)	7.3 (3.3–15.3)	30.9 (20.1–44.2)	4.8 (1.6–13.4)	24.8 (14.3–39.4)	5.3 (1.7–15.2)	0.6 (0.3–1.6)
Southern Yobe	41.1 (28.4–55.0)	58.3 (48.4–67.5)	7.7 (3.8–14.9)	19.4 (13.4–27.2)	4.6 (2.2–9.6)	12.4 (7.8–18.9)	0.9 (0.3–2.8)*	1.5 (0.5–4.4)*
Northern Yobe	28.8 (18.1–42.6)	67.1 (50.3–80.5)	5.5 (2.2–12.9)	30.4 (19.6–43.9)	6.2 (2.3–15.5)	27.3 (16.4–41.8)	2.5 (0.8–7.1)*	0.7 (0.3–1.7)*

**Abbreviation:** CI = confidence interval.

* Difference between round 1 and round 2 was not statistically significant.

The prevalence of diarrhea among children aged <5 years during the preceding 2 weeks and receipt of appropriate treatment (oral rehydration solution or zinc) were analyzed as an indication of morbidity and access to primary care, respectively (Table 3). Prevalence of diarrhea was lower in Borno state regions (range = 14.1%–19.5%) than in Yobe state (range = 23.8%–31.5%) during the first round. In the second round, the prevalence of diarrhea did not change significantly in Yobe but increased significantly in all Borno regions. Among children with diarrhea, less than a third received oral rehydration solution, zinc, or both in either round, and <8% received the appropriate treatment. The proportion of children receiving treatment significantly declined in Southern Yobe and Central Yobe (for zinc only) between survey rounds.

**TABLE 3:** Emergency survey prevalence of diarrhea during the preceding 2 weeks and access to recommended treatment among children aged 0–59 months, by region — Northeastern Nigeria, round 1 (October–November 2016) and round 2 (February–March 2017)

State/Region	Round 1				Round 2			
	Diarrhea prevalence % (95% CI)	Among children with diarrhea % (95% CI)			Diarrhea prevalence % (95% CI)	Among children with diarrhea % (95% CI)		
		ORS	Zinc	ORS and Zinc		ORS	Zinc	ORS and Zinc
**Borno**								
Southern Borno	14.1 (8.9–21.6)*	15.6 (7.6–29.2)	1.3 (0.2–7.4)	0	26.1 (21.4–31.5)*	16.6 (9.2–28.0)	4.3 (1.9–9.4)	1.8 (0.6–5.5)
Central Borno	19.5 (14.7–25.3)*	13.9 (8.3–22.2)	5.0 (2.1–11.1)	4.0 (1.5–10.0)	30.0 (23.9–37.0)*	15.9 (9.5–25.5)	3.8 (1.4–10.0)	2.5 (0.6–10.4)
Maiduguri and Jere	17.9 (12.1–25.7)*	26.1 (16.3–39.2)	4.5 (1.9–10.7)	2.3 (0.6–7.7)	31.7 (26.8–37.0)*	28.4 (19.3–39.8)	9.0 (4.7–16.5)	7.1 (3.8–13.0)
**Yobe**								
Central Yobe	23.8 (18.5–30.1)	15.2 (9.2–24.1)	9.4 (4.3–19.2)*	4.3 (1.7–10.8)	26.5 (20.4–33.7)	12.1 (7.2–19.5)	2.9 (1.2–6.7)*	2.9 (1.2–6.7)
Southern Yobe	31.5 (25.9–37.7)	26.7 (17.9–38.0)*	24.6 (15.0–37.6)*	5.9 (2.3–14.1)*	28.0 (23.0–33.6)	11.4 (7.7–16.6)*	3.3 (1.8–5.8)*	0.8 (0.3–2.6)*
Northern Yobe	30.7 (26.0–36.0)	14.5 (9.1–22.3)	7.5 (2.4–21.4)	0.6 (0.1–4.1)	26.3 (20.0–33.9)	16.1 (7.7–30.4)	3.6 (1.3–9.6)	1.8 (0.4–7.8)

**Abbreviations:** CI = confidence interval, ORS = oral rehydration solution.

* Statistically significant difference between round 1 and round 2.

## Discussion

The prevalence of global acute malnutrition did not exceed the WHO emergency threshold of 15% in either survey (*5*); prevalence either improved (Maiduguri and Jere; Northern and Southern Yobe) or remained stable (Southern and Central Borno; Central Yobe) between rounds. Despite these findings, the crude mortality rate increased significantly in all but two of the surveyed regions (Southern Borno and Central Yobe). Although the study was not designed to detect significant changes in child mortality, an increase in the under-five mortality rate was observed in all regions. Increased mortality in the absence of concurrent increase in acute malnutrition suggests possible causes other than food insecurity, such as increased morbidity and mortality from common childhood illnesses.

The findings from these surveys provide evidence of increases in the use of selected public health interventions. Measles vaccination coverage approximately doubled in several regions of Borno and Yobe after a January 2017 campaign to vaccinate an estimated 4.7 million children, which was recommended after the first survey round. Both the percentage of households that received services during the government sponsored health campaign and the percentage of children who received anthelmintic prophylaxis increased significantly in all regions. Emphasis on active outreach through mobile clinics and household visits, particularly in the November–December 2016 public health campaigns, might have contributed to these increases. Yet receipt of all four public health interventions remains well below target levels, and there has been no improvement in the distribution of fortified cereal.

The percentage of children who received appropriate treatment for diarrhea, assessed as an indication of primary health care service use, was low overall. In both surveys, fewer than one in three children with diarrhea received any treatment, and the proportion of children who received treatment either failed to increase (Borno) or declined (Southern Yobe). Conflict-related destruction has resulted in the loss of two thirds of Borno health facilities (*6*). Southern Yobe, particularly in the local government areas of Gujba and Gulani, experienced increased violence in the months between survey rounds (*7*). Constrained population movement and loss of health care facilities might partially explain the poor access to recommended treatment.

The findings in this report are subject to at least two limitations. First, because of the active conflict, some areas were inaccessible to survey teams, and it is likely that health and nutritional outcomes are worse in these regions. Second, to prioritize rapid data collection, the emergency surveys were designed with relatively small sample sizes that result in estimates with relatively high standard errors (particularly for under-five mortality) and do not allow for disaggregated analysis of subpopulations, such as displaced persons.

The conflict in northeastern Nigeria, which has resulted in mass destruction of health facilities and limited access to treatment, has contributed to emergency levels of mortality throughout the affected region, particularly among children. Ongoing humanitarian activities in Nigeria prioritize interventions known to reduce maternal and child undernutrition and mortality (*8*). However, survey results indicate that substantial gaps in use of these important interventions remain. Until effective interventions are adequately used, they will have limited effect on large-scale mortality, particularly among vulnerable populations including children.

**Summary**

**What is already known about this topic?**

A public health emergency was declared in northeastern Nigeria in June 2016, as rapid assessments conducted in areas newly liberated from Boko Haram control suggested rates of mortality and prevalence of acute malnutrition exceeded emergency levels. Outbreaks of polio and measles have been confirmed, and the Government of Nigeria has expressed concern in response to evidence of acute malnutrition in excess of emergency thresholds. Increased prevalence of acute malnutrition and high rates of mortality are often observed in complex humanitarian emergencies.

**What is added by this report?**

Results from population-representative surveys examining acute malnutrition and mortality conducted in accessible areas in Borno and Yobe states suggest that, although the coverage of major public health interventions, including measles vaccinations, has improved, it remain below targeted levels. All-cause mortality among all age groups (crude mortality) and among children aged <5 years (under-five mortality) was above emergency thresholds in regions of Borno and Yobe states during February–March 2017; this was an increase in the mortality observed during October–November 2016.

**What are the implications for public health practice?**

Increasing mortality rates suggest a need for enhanced efforts to improve receipt of ongoing lifesaving interventions, including treatment of common childhood illnesses in conflict-affected areas. Use of preventive services such as measles vaccination improved in the months after the emergency declaration; however, treatment services have not. Without efforts to scale up multisectoral interventions targeted at reducing malnutrition and morbidity among children throughout accessible regions of northeast Nigeria, limited impact on mortality can be expected.
